# Chromosomal barcoding as a tool for multiplexed phenotypic characterization of laboratory evolved lineages

**DOI:** 10.1038/s41598-018-25201-5

**Published:** 2018-05-03

**Authors:** Leonie Johanna Jahn, Andreas Porse, Christian Munck, Daniel Simon, Svetlana Volkova, Morten Otto Alexander Sommer

**Affiliations:** 1 Novo Nordisk Foundation Center for Biosustainability, Technical University of Denmark, DK-2800 Kongens Lyngby, Denmark; 2 Present Address: Department of Systems Biology, Columbia University, New York, NY USA

## Abstract

Adaptive laboratory evolution is an important tool to evolve organisms to increased tolerance towards different physical and chemical stress. It is applied to study the evolution of antibiotic resistance as well as genetic mechanisms underlying improvements in production strains. Adaptive evolution experiments can be automated in a high-throughput fashion. However, the characterization of the resulting lineages can become a time consuming task, when the performance of each lineage is evaluated individually. Here, we present a novel method for the markerless insertion of randomized genetic barcodes into the genome of *Escherichia coli* using a novel dual-auxotrophic selection approach. The barcoded *E*. *coli* library allows multiplexed phenotyping of evolved strains in pooled competition experiments. We use the barcoded library in an adaptive evolution experiment; evolving resistance towards three common antibiotics. Comparing this multiplexed phenotyping with conventional susceptibility testing and growth-rate measurements we can show a significant positive correlation between the two approaches. Use of barcoded bacterial strain libraries for individual adaptive evolution experiments drastically reduces the workload of characterizing the resulting phenotypes and enables prioritization of lineages for in-depth characterization. In addition, barcoded clones open up new ways to profile community dynamics or to track lineages *in vivo* or *situ*.

## Introduction

Lineage tracking is a valuable tool used to answer many fundamental biological questions regarding the evolutionary forces and principles behind adaptation processes^1^. Tracking of lineages resulted in the identification of key parameters for evolutionary dynamics^2^, shed light on the deterministic character of evolution^3^, deepened our knowledge in dynamic genetic interactions^4^ and helped us to characterize genetic functions^5–7^ as well as specific mutations in more detail in direct competition experiments^8^ or complex environments such as the gut^9–11^.

Different methods have been utilized to track the population dynamics of co-existing lineages. The first studies applied light microscopy to track cells^12,13^. Later fluorescent tags were used in microscopy to follow specific phenotypes over time^14,15^, during development *in vivo*^16–18^, in cancer models^17^ or to trace individual lineages of cells in a population^19,20^. Fluorescent tags have also been used for evolution experiments^2^ and for allele tracking with qPCR^8^. More recently, genetic approaches have been used for lineage tracking. Whole genome sequencing enabled researchers to follow the evolution of individual MRSA strains in a human host during antibiotic treatment^21^, to characterize the colonialization of the infant gut^10,11^ or to perform multiplexed phenotyping of different gut microbiome species^22^. In addition, the insertion of genetic elements such as microsatellite regions^23^ or genetic barcodes^3^ has broadened our understanding of evolutionary dynamics. While whole genome sequencing is needed to track unmodified bacterial strains in *in situ*, tracking genetically barcoded clones by amplicon sequencing provides a cheaper and faster alternative when the strains can be genetically manipulated.

Here, we present a novel method describing the markerless integration of genomic barcodes at a specific location in the genome of *E*. *coli* without inserting selection markers or altering the genomic sequence except for the additional nucleotides of the barcode. We created a library composed of more than 400 clones carrying unique 12-nucleotide barcodes, that is available upon request, and used a subset of these clones in an adaptive laboratory evolution (ALE) experiment towards antibiotic resistance. Finally, we showed, as a proof of concept that the multiplexed phenotyping enabled through the barcoding scheme presented here, correlates well with traditional phenotyping methods based on individual clones and allows deeper insights into the population dynamics that would not be detected by traditional growth assays.

## Results

### Dual-auxotrophic selection allows for markerless insertion of genetic barcodes

**Figure 1:**
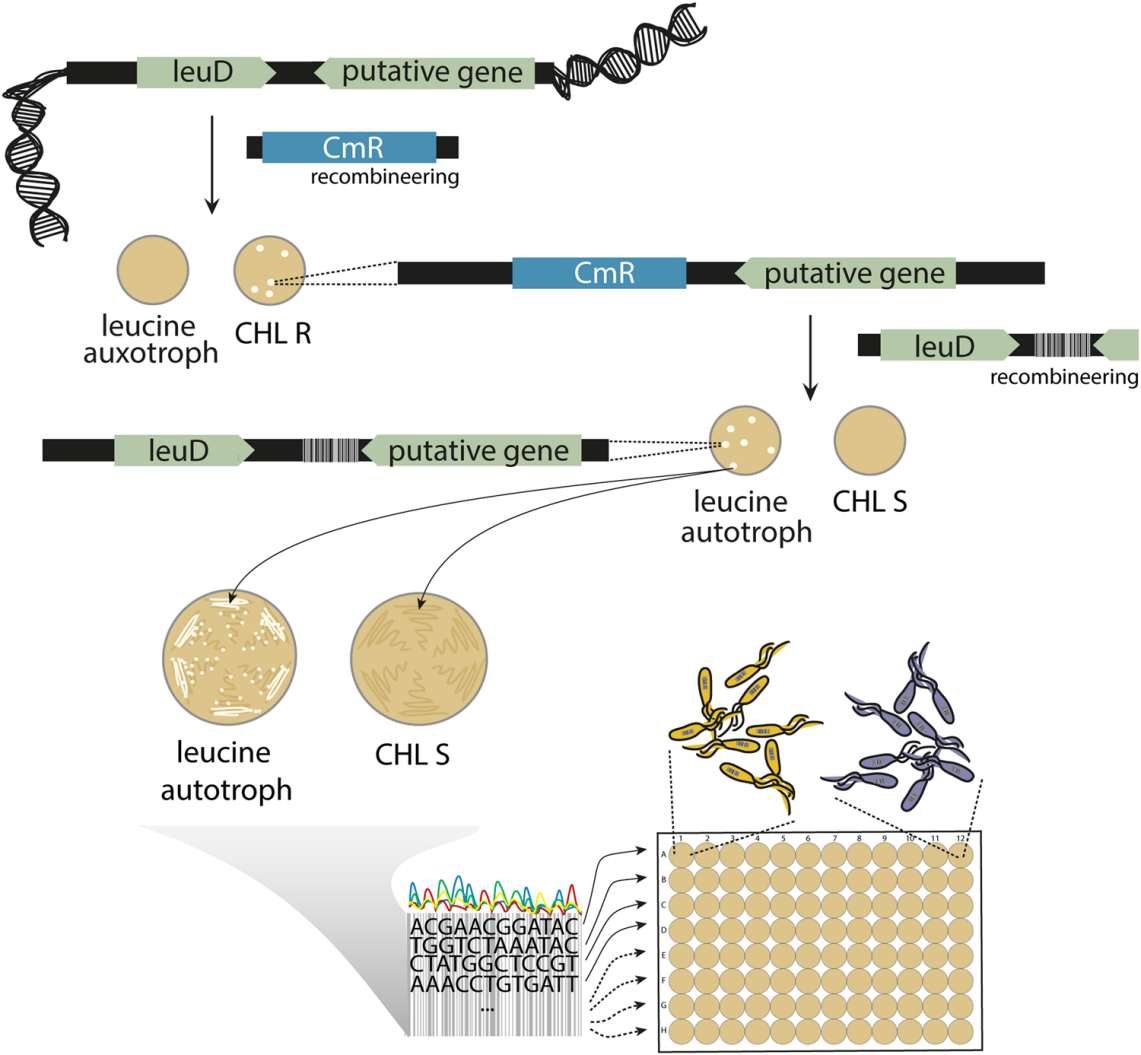
Cloning strategy for creating the barcoded library. *leuD*, part of the leucine operon and essential to produce leucine, was replaced with a chloramphenicol resistance gene through recombeneering^51^. Clones were screened for leucine auxotrophy and chloramphenicol resistance (CHL R). In a next step the replacement was reverted using the native *leuD* gene attached to a random 12-nucleotide long genetic barcode. Clones were selected when they were leucine autotroph and chloramphenicol sensitive (CHL S). Barcode integration was confirmed via Sanger sequencing. A library of 445 uniquely barcoded clones was compiled and cured from the recombeneering plasmid.

To develop a tool for elucidating the dynamics of clonal populations we engineered a library of uniquely barcoded *E*. *coli* strains. As selection markers, especially those that confer antibiotic resistance, could potentially interfere with our interest of studying *de novo* antibiotic resistance mutations, we developed a protocol that allows the marker-free insertion of genetic barcodes into the genome of *E*. *coli*. Insertion of the unique barcodes occurred in a two-step process; first *leuD* of the leucine operon was knocked out with a chloramphenicol resistance gene resulting in a leucine auxotroph and chloramphenicol resistant clone. In the second step the *leuD* gene was reintroduced with a fragment containing a random 12-nucleotide barcode removing the chrolamphenicol marker and restoring leucine autotrophy (Fig. 1). The fragment was created with a primer containing an overhang of 12 random nucleotides allowing us to introduce up to 4^12^ unique barcodes in a single step. We then selected clones that were chloramphenicol sensitive and leucine autotroph. A region including the barcode and part of *leuD* was sequenced to confirm the correct insertion of the *leuD* gene and the specific sequence of each barcode. From the sequencing data we compiled a library composed of 445 clones each harboring a unique barcode at the same position in the genome (SI Table 1).

### Evolution of antibiotic resistance using uniquely barcoded parallel evolving lineages

ALE experiments are commonly applied to study the evolution of antibiotic resistance^24–29^. To illustrate the potential of a library with unique barcodes that allow the tracking and comparison of evolving populations, a subset of the library was used in an antibiotic ALE experiment.

**Figure 2:**
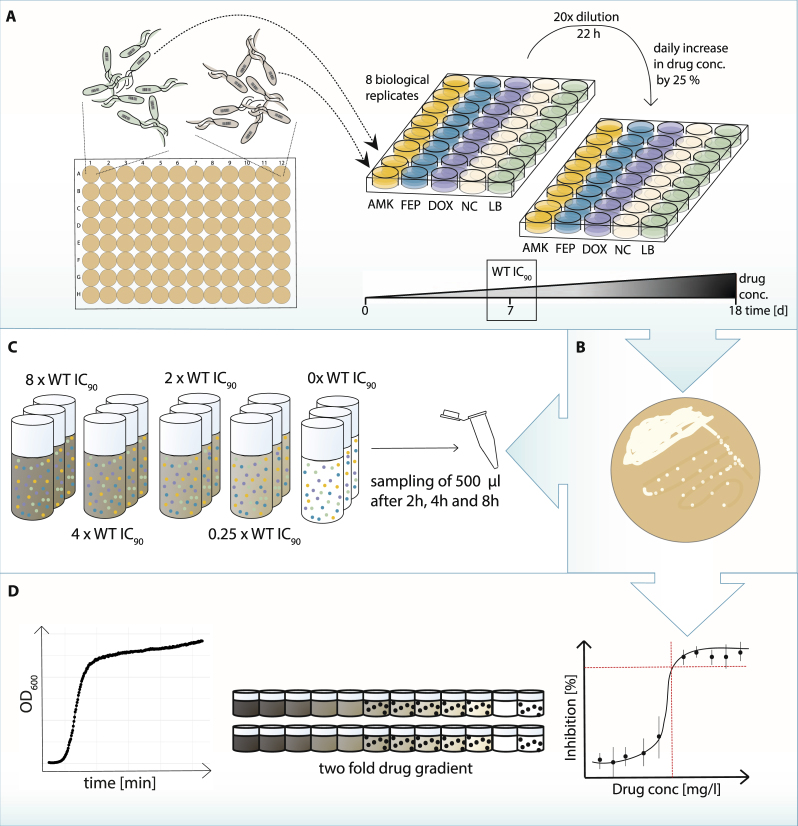
Workflow of the adaptive laboratory evolution (ALE) and subsequent phenotyping. (**A)** ALE towards amikacin (AMK), cefepime (FEP) and doxycycline (DOX) resistance. Uniquely barcoded *Escherichia coli* K12 clones were adapted to AMK, FEP, DOX and the media (LB) for 18 days in 8 replicates. Each replicate carried a unique chromosomal barcode. The populations were transferred every 22 hours with a 20-fold dilution to an increased drug concentration of 25%. The drug concentration at which the OD_600_ of the WT was 10% of the OD_600_ of the positive control before the evolution experiment was started (WT IC_90_) was reached on the 7^th^ day of the evolution experiment. The drug concentration of the last day of the experiment was more than 10-fold higher than the WT IC_90_. (**B)** After the completion of the ALE each lineage was streaked on LB and a single colony was chosen for further experiments. (**C)** Competition experiment of evolved barcoded clones in different antibiotic concentrations. Outgrown cultures of all clones were mixed with equal volumes and used to inoculate media with five different drug concentrations for each antibiotic, as well as without antibiotic (in triplicates). Samples were taken after 2, 4 and 8 hours. (**D)** Traditional phenotyping methods such as growth kinetics in different antibiotic concentrations and resistance level determination in a drug gradient were performed for each clone.

We adapted 8 parallel lineages to 3 different antibiotics, the aminoglycoside amikacin (AMK), the tetracycline doxycycline (DOX) and the beta-lactam antibiotic cefepime (FEP) as well as to lysogenic broth (LB) medium as a control (Fig. 2A). The 3 drugs represent 3 major antibiotic drug classes with different mechanisms of action, including both bactericidal as well as bacteriostatic drugs. For the ALE experiment, we inoculated each replicate with a uniquely barcoded clone. Therefore, 32 different genetically barcoded lineages were required and randomly selected from the barcoded library. The ALE was carried out for 18 days and was started at sub-inhibitory antibiotic concentrations. The drug concentrations were increased by 25% at every daily transfer as previously described^29^. The IC_90_ of the wild type (WT) was reached on the 7^th^ day of evolution. The IC_90_ is defined as the drug concentration at which the optical density (OD_600_) of the tested strain is 10% of the OD_600_ of the WT grown without exposure to the respective drug^8^. On the last day of the evolution experiment, lineages were exposed to drug concentrations exceeding the WT IC_90_ by at least 10-fold. Exact drug concentrations for each day and each drug can be found in SI Table 2. A single colony was isolated at the end of the evolution experiment for each replicate lineage (Fig. 2B).

### Traditional phenotyping methods reveal cross-resistance between DOX and FEP evolved lineages

**Figure 3:**
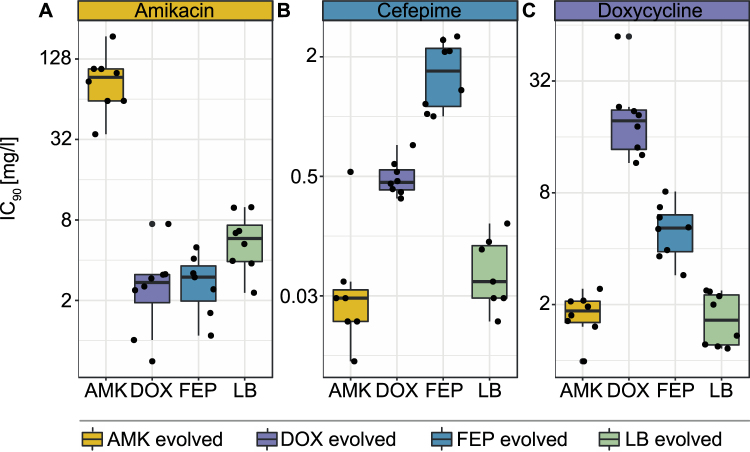
Antibiotic susceptibility of evolved isolates. Inhibitory drug concentrations (IC_90_) of **(A)** amikacin, **(B)** cefepime and **(C)** doxycycline after the adaptive laboratory evolution (ALE) experiment. The IC_90_ was measured for all 8 biological replicates after the adaptive evolution experiment for all 3 drugs. Clones adapted to amikacin (AMK), cefepime (FEP), doxycycline (DOX) and medium (LB) are displayed in yellow, blue, purple and green, respectively.

Traditionally the resistance level of a single clone is determined by measuring the minimal inhibitory concentration in microbroth dilutions. We measured the IC_90_ of all lineages towards the 3 antibiotics using 2-fold microbroth drug dilutions. All lineages evolved high-level resistance to the drug they were exposed to during the evolution experiment (Fig. 3). The IC_90_ values of the media adapted WT were comparable to the IC_90_ values of the ancestor WT, measured before the experiment started, as the average of the media adapted IC_90_ values were less than 2-fold different from the original IC_90_ values (SI Table 3). Yet, a variability among biological replicates was observed that might be accounted by media adaptations potentially altering the susceptibility profiles^30^. The most resistant clones for each drug were ~50 (AMK), ~60 (FEP) and ~30 (DOX) fold more resistant compared to the WT, while the least resistant clones were only 5-fold more resistant than the ancestor (Fig. 3). This highlights that adaptive evolution towards these drugs can follow a wide range of evolutionary trajectories.

Lineages that become resistant to one antibiotic often display either resistance or increased susceptibility to other antibiotics^25,26,31^. These phenotypes are referred to as collateral resistance and sensitivity, respectively. In this study, clones evolved to FEP displayed an elevated resistance towards DOX and *vice versa*. DOX and FEP evolved lineages were on average slightly more susceptible towards AMK than the media adapted WT (Fig. 3). Mutations increasing drug efflux through AcrAB are commonly found in antibiotic resistant bacteria^8,26,32^. While increased efflux confers resistance towards many different antibiotics, it is not effective against aminoglycosides^26^. An interaction between the membrane potential, crucial for the uptake of aminoglycosides, and AcrAB mediated efflux has previously been documented to explain the collateral sensitivity of aminoglycoside resistant strains towards other antibiotics such as DOX and FEB^26^. This well explained mechanism of collateral sensitivity was also observed in this study between cells adapted to AMK tested in FEP. Yet, it does not explain the observed collateral sensitivity between DOX and FEP evolved lineages towards AMK. We speculate that cells adapted to a high AcrAB mediated efflux are also adapted to sustain a high membrane potential to fuel the high efflux rate. This in turn could result in a higher uptake of aminoglycosides, explaining the slightly increased susceptibility of DOX and FEP evolved lineages towards AMK.

**Figure 4:**
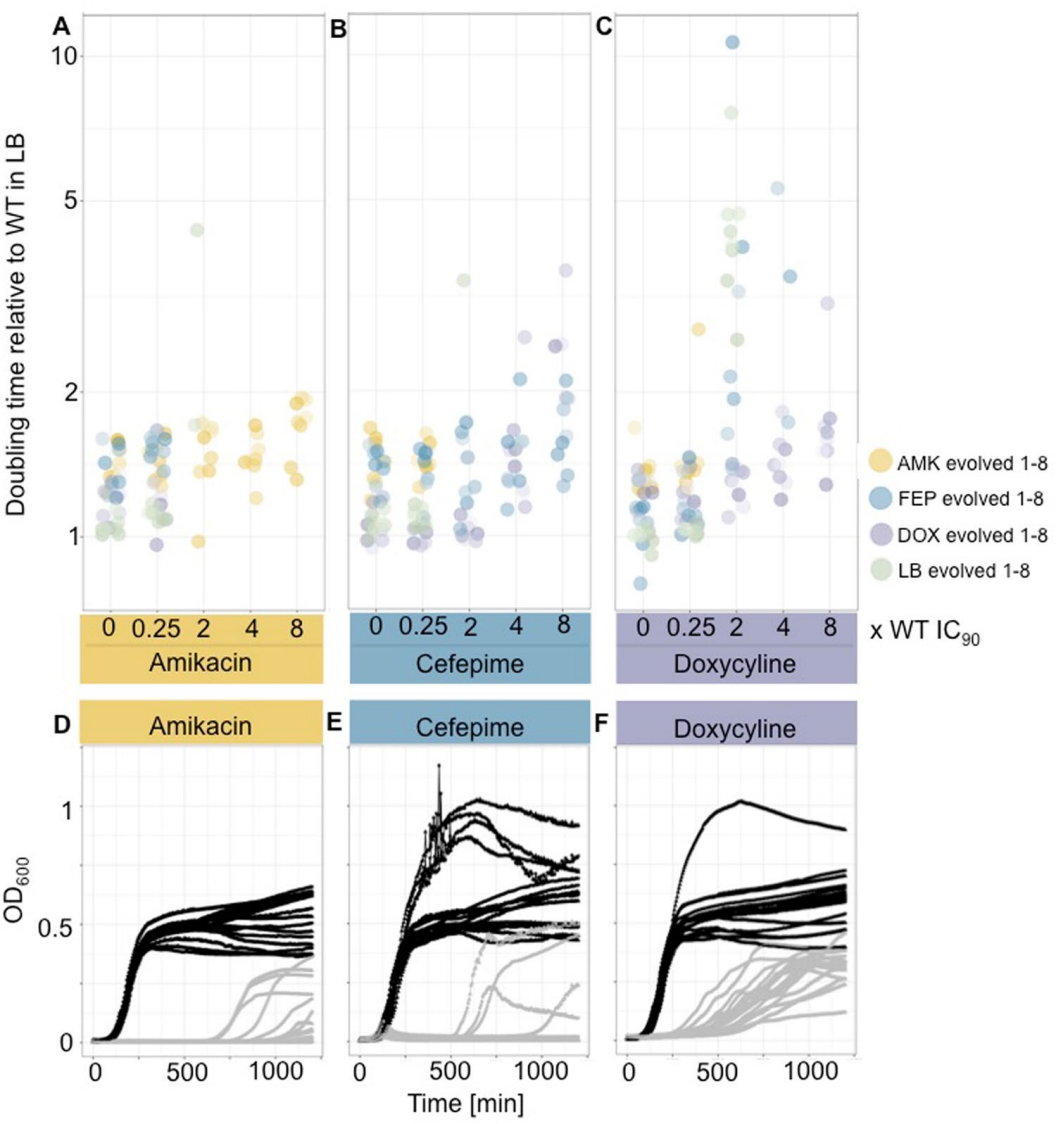
Growth properties of evolved clones. Doubling time of evolved lineages in different concentrations of **(A)** amikacin, **(B)** cefepime and **(C)** doxycycline. The doubling time of the evolved lineages was measured without antibiotic (0 × WT IC_90_), in sub-inhibitory drug concentrations (0.25 × WT IC_90_) and in 2, 4 and 8 fold of the WT IC_90_. The doubling time was normalized to the ancestor WT grown in antibiotic free LB medium. Clones adapted to AMK, FEP, DOX and LB are displayed in yellow, blue, purple and green, respectively. The intensity of the color increases with the resistance level towards the drug they were evolved to. Growth curves of media adapted clones in **(D)** amikacin, **(E)** cefepime and **(F)** doxycycline. The growth curves of all 8 biological and 2 technical replicates of the media adapted clones reveal a huge difference in the growth phenotype in sub-inhibitory drug concentrations (black lines) and 2-fold WT IC_90_ drug concentrations (grey lines).

Growth-rate measurements are another standard experiment used as a proxy for the fitness in the characterization of mutants resulting from ALE experiments^33–35^. We assessed the growth rate of the evolved clones in a sub-inhibitory concentration as well as in 2, 4 and 8 fold of the WT IC_90_ drug concentrations for all three drugs in 2 technical replicates (Fig. 4A–C). The doubling time was normalized with the doubling time of the ancestor (WT) growing in LB medium without antibiotics (Fig. 4A–C, SI Table 4). The medium adapted lineages had a similar doubling time compared to the ancestral WT (Fig. 4A–C). The drug-evolved clones had slower growth rates compared to the media evolved lineages (SI Tables 4 and 5, Mann-Whitney-U-test, p < 2.2e^−16^). All lineages were capable of growing in the sub-inhibitory drug concentrations of all 3 drugs; indicating the absence of strong evolved collateral sensitivity. As a general trend it can be noticed that the doubling time increases with the antibiotic concentration; also for the clones that were adapted to the respective drug. We observed some exceptional clones (biological replicates 1 and 6 of AMK evolved lineages, biological replicate 2 of FEP evolved lineages and biological replicate 6 of DOX evolved lineages, SI Table 4) with a stable doubling time independent of the drug concentration for all 3 drugs. For AMK and DOX adapted lineages those clones were among the most resistant ones while no correlation between the resistance level and the growth kinetics could be established for FEP evolved clones. In the 2-fold WT IC_90_ drug concentrations some media adapted lineages are unexpectedly able to grow. However, often only one of the technical replicates was able to grow, lag phase was prolonged and the final OD levels were reduced compared to growth in sub-inhibitory concentrations (Fig. 4D–F). These growth patterns could be explained by genotypic differences in the media adapted lineages that could potentially alter their susceptibility patterns^30^ or by spontaneous mutations arising during the growth rate measurements. Lineages evolved to DOX and FEP displayed a cross-resistance phenotype as also indicated by the IC_90_ measurements.

### Barcoding allows for multiplex phenotyping of evolved strains

By pooling the clones for multiplexed phenotyping based on the barcodes it is possible to reduce the workload needed for the characterization of single clones; especially, when many different conditions are analyzed. We hypothesized that outcomes of the multiplexed analysis would be comparable to our data obtained for each clone tested individually. To test this hypothesis, outgrown overnight cultures of all clones were mixed in equal volumes. A sample of this mixture was frozen as time point 0. Of the remaining mixture, 5 ml of media either without antibiotic, with sub-inhibitory drug concentrations, or 3 different drug concentrations above the WT IC_90_ were inoculated in triplicates for each antibiotic (Fig. 1C). Samples were taken after 2, 4 and 8 hours.

**Figure 5:**
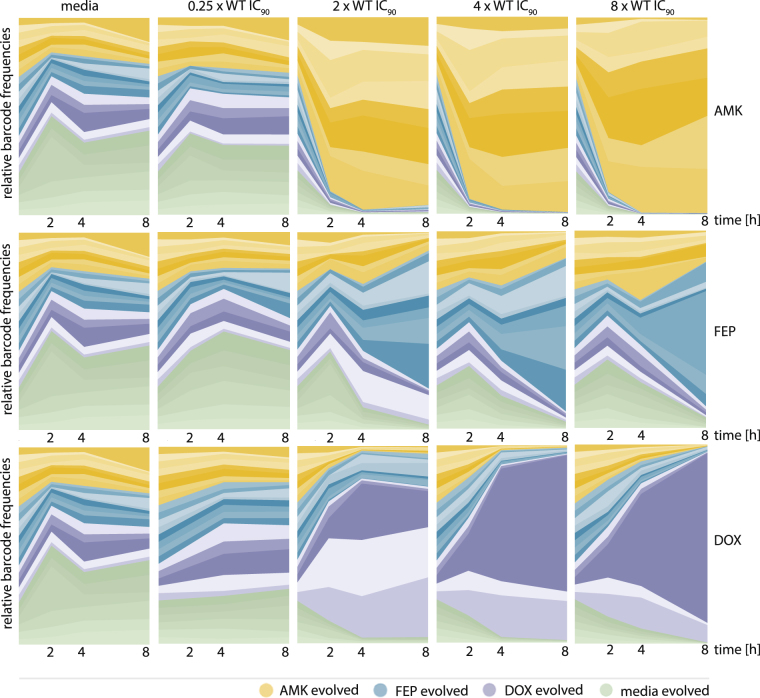
Relative barcode frequencies for each lineage over time in different concentrations of amikacin (AMK), cefepime (FEP) and doxycycline (DOX). Each column represents the different conditions: lineages grown in the media without drug (media), media containing sub-inhibitory drug concentrations (0.25 WT IC90) or three different concentrations above the WT IC_90_ (2×, 4× and 8× WT IC_90_). The three rows represent the three different drugs. Each plot displays the relative barcode abundance of each lineage over time. Clones adapted to AMK, FEP, DOX and media are displayed in yellow, blue, purple and green, respectively. The intensity of the color increases with the resistance level of each clone towards the drug they were evolved to.

The barcode was amplified and deep sequenced for the whole population subjected to each condition, replicate and time point, and the barcode frequencies were normalized to time point 0. The average of the replicates was calculated. In the antibiotic free medium, the LB medium adapted WT appears to be dominating. However, its fitness advantage was not pronounced enough to outcompete the other clones within an 8-hour competition experiment (Fig. 5). The fitness benefit of the WT is only detected in conditions without selection. Under selection clones adapted to the respective drug are clearly favored over time (Fig. 5). The time needed until the drug-adapted lineages dominate the population varies for each drug. In AMK AMK-evolved clones take over the population already within the first 2 hours of growth while 8 hours are not enough for the FEP-adapted clones to fixate despite FEP treatment. The frequencies of the biological replicates adapted to the same drug varied when exposed to different drug concentrations. The most resistant clone for DOX is dominating the population after 8 hours only when the population was exposed to 4 and 8 fold WT IC_90_ drug concentrations but not at 2-fold (Fig. 5). All lineages adapted to AMK and FEP were more resistant than 8 fold of the WT IC_90_ of the respective drug. Drug conditions below the resistance level seem not necessarily to select for the mutant with the highest IC_90_ value and other fitness aspects appear more influential under these conditions.

Interestingly, we observed, in the populations exposed to inhibitory drug concentrations of FEP, that DOX adapted lineages are outcompeted more than AMK adapted lineages. This result stands in contrast to the individual growth kinetics of the clones that did not allow AMK adapted clones to grow under these conditions while DOX adapted lineages were able to grow also in higher FEP concentrations (Figs 4A–C and 5). Consistent with the ability of FEP adapted lineages in the single growth kinetic measurements, FEP adapted lineages are also able to grow in low inhibitory concentrations of DOX in the pooled populations (Fig. 5).

In summary the IC_90_ determines largely weather a lineage is able to grow under a specific drug regime. However, other factors influencing the fitness, e.g. the costs of adaptations as well as the interaction with other lineages, decide about the degree of growth under the respective condition.

### The results from traditional and multiplexed phenotyping methods are positively correlated

**Figure 6:**
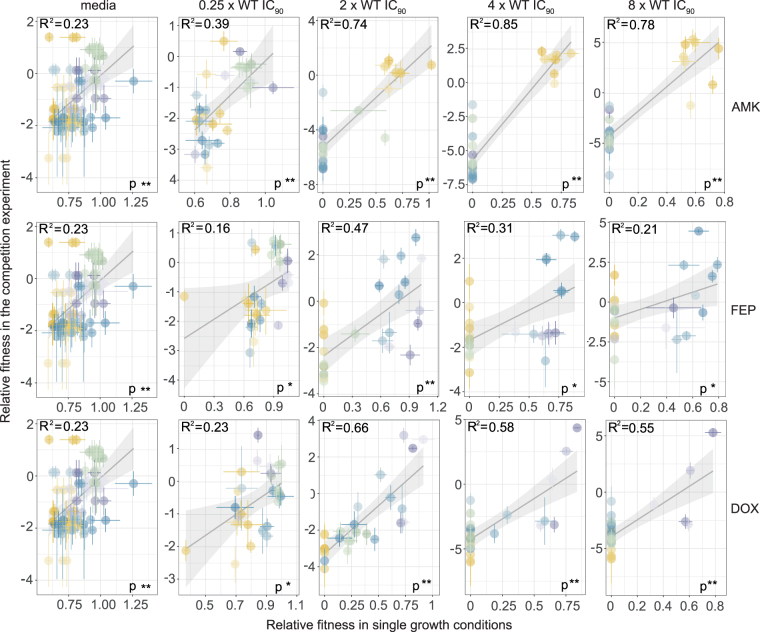
Significant positive correlation of the fitness calculated based on individual and pooled phenotyping. The different columns represent the different conditions: lineages grown in the media, sub-inhibitory drug concentrations or three different concentrations above the WT IC_90_. The three rows represent the three different drugs. Each plot displays relative fitness calculated from the competition experiment versus the relative fitness derived from individual growth measurments. The coefficient R^2^ and the p-value of a Pearson’s product-moment correlation are provided for each linear regression model (*P < 0.05, **P < 0.001). Clones adapted to amikacin (AMK), cefepime (FEP), doxycycline (DOX) and media are displayed in yellow, blue, purple and green, respectively. The intensity of the color increases with the resistance level towards the drug they were evolved to.

Finally, we tested our hypothesis that pooled and individual clone characterization results in similar outcomes by correlating the fitness of the clones based on the pooled and individual experiments. In the linear regression model we used the fitness derived from the single clone measurements as the predictor and the fitness calculated from the barcode frequencies as a response variable. We find a significant (P < 0.05, Pearson’s product-moment correlation), positive correlation of the fitness in all conditions after 8 hours of growth (Fig. 6). However, the variability is fairly high and the R^2^ varies from 0.16–0.85 dependent on the drug and condition. The variability in the linear regression models for FEP exposed lineages is especially high. As described before we also observed a discrepancy in the phenotypes of the pooled analysis and the single clone measurements for DOX and AMK adapted lineages, which might account for the high variance. The variability would likely decrease by prolonging the competition experiment and therefore allowing the fittest clones more time to outcompete the other lineages.

Due to the high variability in the correlation between growth rates and competition outcomes, a second evaluation of the multiplexed phenotyping was performed. We ranked the fitness of each clone for each drug and condition from both multiplexed as well as individual phenotyping (SI Table 6). Under conditions with selection (2×, 4× and 8× WT IC_90_) the best performing clone obtained from the individual growth measurements was among the 5 best performing clones of the competition experiment in the same drug condition. Consequently, picking the best 5 clones for in depth characterization will likely include similar mutants regardless of the fitness assay used.

In conclusion, multiplexed phenotyping of a pooled set of clones correlates with traditional techniques to characterize mutants after ALE experiments and allows identifying the best performing clones under selecting conditions. The barcoded library, created for this study, is consequently a valuable resource for high-throughput ALE experiments as it reduces the workload of subsequent characterization of mutants drastically.

## Discussion

Genetically barcoded strains are highly useful to study the population dynamics, also those that result from random processes e.g. population bottlenecks and genetic drift that are not driven by mutant selection, as well as evolutionary processes of heterogenic populations. In addition, barcoded clones can be used to multiplex phenotyping of a larger number of similar strains, e.g. those resulting from ALE experiments. Different strategies for genetic barcoding exist. Yet, to our knowledge all of them depend on the insertion of selection markers or other modifications of the genome^1^. Here, we present a novel approach for inserting genetic barcodes in the genome of *Escherichia coli* without any additional modifications. An unaltered genetic background can be important for example when different antibiotic resistant strains or clones shell be compared in their response to various antibiotics. Moreover, if barcoded clones are used for production strain improvements for example by ALE experiments or transposon mutagenesis and subsequent multiplexed phenotyping, an unaltered genetic background is desired as many production strains already carry genetic alterations and additional selection markers could limit the number of available markers. Additionally, selection markers might burden the cell, which could result in reduced growth rates. Therefore, a strain with as little genetic changes as possible is a desired starting material. To achieve the markerless insertion of genetic barcodes we used *leuD* as selection marker. Even though other selection systems using *sacB*, *galK* or *thyA* are well established they have a narrow compatibility with high escape rates^36^, which we did not observe for the native metabolic *leuD* gene. Auxotrophic selection markers can be established in every organism and belong to the standard tools for genome editing in yeast^37,38^, highlighting their potential role in genetic engineering of *E*. *coli* or other microbes.

Multiplexed phenotyping reduces the workload by screening for the fittest mutant under different conditions. While genetic barcodes might not be necessary when genetically distant lineages are used^22^, lineages resulting from ALE experiments might differ only in a few nucleotide polymorphisms^29^, wherefore genetic barcodes are required to distinguish clones from one another. We could show that multiplexed phenotyping results correlate with traditional phenotyping methods based on individual measurements. Small discrepancies were observed for clones adapted to AMK and DOX exposed to FEP. It should be noted that a shortcoming of the correlation analysis is that the growth rate measurements and pooled competition experiments characterize different aspects of bacterial fitness. While the doubling time is a widely accepted proxy for bacterial fitness^38,39^ it is calculated by using only a fraction (steepest part of the growth curve) of the whole growth curve that is taken into account in the competition experiment. Different growth dynamics, especially for strains subjected to antibiotic pressure, including different durations and behaviors of lag, log and stationary phases (Fig. 4 D–F) and interactions with other strains influence the outcome and resolution of competition experiments and might be especially important for physiologically challenged mutants. In fact, we observed altered growth dynamics for some mutants and conditions, especially under exposure to FEP suggesting that growth rate measurements might be a better proxy for fitness under stress free conditions or for mutants that are not physically challenged. Additionally, the presence of other lineages can influence the performance of each clone in a given environment. By pooling all lineages the conditions are less controlled compared to individual growth kinetics, which might be attributed to nutrient availability, the antibiotic exposure and the overall composition of the media changing with metabolites produced from the other cells or through cellular material from lysed cells. Metabolic shifts can result in different tolerance levels for antibiotics^40,41^, suggesting that the production of metabolites by a subpopulation might influence the metabolism of susceptible clones increasing or decreasing their tolerance towards a certain antibiotic^41^. For example, it has been found that resistant lineages can produce the compound indole that might increase the tolerance of susceptible clones in the population^42^. Those complex interaction dynamics in bacterial populations are more likely to be detected in pooled phenotyping setups and might more accurately reflect the fitness of bacteria in their natural habitats compared to measurements performed on individual clones.

Multiplexed phenotyping may not completely substitute traditional phenotyping methods. Yet, it can reduce the workload and help to identify promising lineages for in-depth characterization. In addition, it opens ways to study population dynamics, to detect cross-resistance and collateral sensitivity and to identify the fittest mutant in various conditions including challenging environments like urine, wastewater or *in vivo* models. Furthermore, clones adapted to the same condition can be compared in competition experiments, which can help to foster our understanding of the relation between genotype and phenotype.

In conclusion adaptive evolution using barcoded strains harbors a big potential in reducing workload of characterization of evolved strains and opens up the opportunity for a more complex and detailed analysis of the population dynamics of co-existing lineages.

## Materials and Methods

### Creation of barcoded library

The first step towards the integration of 12-nucleotide long genetic barcodes in *Escherichia coli* was to amplify the chloramphenicol resistance gene from a pZ cloning vector^43^ with the following primers: KO_leuD_F: AGGTTAAAGACGTTTGATGACGT-GGACGATAGCGGAAAGCCCGGTCATTTAGTGCTTGGATTCTCACC and KO_leuD_R: TGTG-ACCGGACATTTCGCCGACATTCGCAACATTAAATAAGGAGCACACCCTCTGGTAAGGTTGGG, flanking the beginning of the *leuD* gene and the area downstream of the gene. The PCR product was purified with the NucleoSpin Gel and PCR Clean-up kit by Machery-Nagel according to the manufacturers instructions and used for electroporation in MG1655 carrying the pSim6 plasmid^44^ allowing the chloramphenicol resistance gene to replace *leuD* through recombeneering. Cells were recovered in lysogeny broth (LB) overnight and plated on LB agar containing chloramphenicol (30 μg/ml) the next day. Colonies were picked and streaked on LB, LB containing ampicillin (100 μg/ml), LB containing chloramphenicol and on Synthetic Complete media (SC) lacking leucine. One clone that grew on all plates except the SC plate lacking leucine, was used to inoculate 5 ml LB containing ampicillin and chloramphenicol. It was used for recombination with another purified PCR product created with primers: Re_tag_leuD_F: GTTATTTCTGTTGTCGCA-TTATTTTTAACCGCAAAGGTTAAAGACGTTTGnnnnnnnnnnnnATGACGTGGACGATAGCGG and Re_leuD_R: TGAACAACGACCGTCTGAATCC using MG1655 WT DNA as a template. The forward primer is carrying the 12-nucleotide long random barcode. After recombination, cells were recovered overnight and plated on LB containing chloramphenicol. More than 1000 colonies were picked and streaked for isolated colonies on LB containing chloramphenicol as well as on SC lacking leucine. Isolated colonies that were sensitive to chloramphenicol and leucine autotrophs were picked and used to inoculate 96-well microtiter plates containing 150 μl of LB. 1 μl of the overnight culture was used for a colony PCR amplifying the barcoded area with the following primers: Re_leuD_F: GGATTTAATGCCTGGAAGAGC and Re_leuD_R. The PCR products were purified using the ZR-96-well DNA Clean-up kit by Zymo Research according to the manufacturers instructions. The purified products were used for Sanger sequencing using the Mix2Seq kit by Eurofins Genomics according the manufacturers instructions including Re_leuD_F as primer and the Sanger sequencing services by BaseClear. Clones that contained the *leuD* gene as well as an unique barcode were transferred to a new 96-well plate containing LB and the position was noted together with the barcode sequence. The clones were frozen in 25% glycerol stocks at −80 °C. Subsequently; these clones were grown at 42 °C to cure them from the pSim6 plasmid^44^. They were streaked on LB agar containing ampicillin and the same toothpick was used to inoculate a well in a 96-well plate. This was repeated until no colonies could be identified on the LB agar containing ampicillin. The final 96-well plate containing the cured clones were replicated into LB containing ampicillin using a 96-well pin-replicator and no growth was detected. The final barcoded library compasses 445 clones each with a unique barcode. A list of all barcodes can be found in SI Table 1. The cured strains were frozen in 25% glycerol stocks at −80 °C.

### Adaptive laboratory evolution with barcoded strains

A subset of the barcoded library, 32 clones, were used for an ALE experiment. The clones were exposed to either AMK, FEP, DOX or LB in 8 biological replicates. Another 8 wells contained LB media but were not inoculated to serve as a negative control. All negative control wells stayed uncontaminated throughout the experiment. The antibiotic exposure started at sub-inhibitory drug concentrations and was raised every day by 25%^29^ reaching the WT IC_90_ on the 7^th^ day of evolution and ending after 18 days with a >10 fold higher drug concentration than the WT IC_90_. The exact drug concentrations for each day of the experiment can be found in SI Table 2. The experiment was performed in a volume of 1 ml in 96-deep well plates by Almeco. All plates were prepared with fresh drug stocks prior to the experiment and kept at −20 °C. Every 22 hours, the optical density was measured at a wavelength of 600 nm (OD_600_) in an ELx808 Absorbance reader (BioTek), 50 μl of the cultures were frozen at −80 C in 25% glycerol and 50 μl were used to inoculate a fresh plate with a higher drug concentration. The new plate was defrosted on the day of usage and preheated to 37 °C. The inoculated plates were incubated at 37 °C and 900 r.p.m. Each lineage was streaked on LB agar after completion of the ALE and an isolated colony was obtained for further experiments.

### IC_90_ determination

Each isolated colony was grown in LB overnight and a 96-well pin-replicator (Almeco) was used to inoculate the broth microdilutions in technical replicates. A 2-fold drug gradient was used for each antibiotic. Growth was normalized with the average blank values from the negative controls and the average growth in media without antibiotic as described before^8,29^. Dose response curves were fitted to the data using R^45,46^ and the concentration at which the OD_600_ was 10% of the OD_600_ of the positive control, was determined^8,29,47^. All IC_90_ values are presented in SI Table 7 along with MIC values, determined according to standard protocols^48^.

### Growth rate measurements

96-well microtiter plates containing 200 μl LB and different antibiotic concentrations (SI Table 8) were inoculated with a 96-well pin-replicator (Almeco) with cells in exponential growth phase. On each plate, 4 wells were reserved to the ancestor WT growing in LB and at least 4 wells served as negative controls. The OD_600_ was measured every 5 min for 20 hours. The plates were kept at 37 °C and 650 r.p.m. The data was analyzed with R^46,49^, calculating the doubling time based on the steepest part of the growth curve^29^. The doubling time was normalized to the doubling time of the ancestor WT in LB.

### Fitness calculation based on growth kinetics

The fitness was calculated based on the doubling time. The average doubling time of the ancestor WTs growing in LB was divided by the doubling time of each lineage as described before^4^. The average and standard derivation of the replicates was calculated in RStudio (0.99.467).

### Competition experiment

For multiplexed phenotyping all clones were grown in LB overnight and mixed in equal volumes. An aliquot of this starting mixture was frozen as time point 0. 50 μl of the mixture were used for inoculation of every triplicate of 4 different (one sub-inhibitory and three inhibitory) drug concentrations for each antibiotic in a total volume of 5 ml. The exact drug concentrations are provided in SI Table 8. The tubes were incubated at 37 °C and 1200 r.p.m. After 2, 4 and 8 hours 500 μl of the cultures were sampled and frozen at −20 °C. Depended on the drug condition and time point varying amounts of the samples (0.5–250 μl) were used for further analysis. 0.5 μl of the samples taken from 0 and 8 hours were mixed with 12.5 μl water. For the other time points varying amounts were spun down so that a tiny pellet was visible in the tube. The pellet was resuspended in 13 μl of autoclaved miliQ water. All samples were incubated at 99 °C for 7 minutes. A 15-cycle PCR was performed directly on the cells using the following primers 40-fold diluted: Fwd_Primer: TCGTCGGCAGCGTCAGAGTGTATAAGAGACACAATGACCGGGCTTT-CCGC and Rev_Primer: GTCTCGTGGGCTCGGAGATGTGTATAAGAGACAGGGATGCTATGG-TTTCAGG with homology to the NEBNext Multiplex Oligos for Illumina (Index primer sets A and C) (New England BioLabs). The PCR product was directly used for indexing PCR with 20 cycles using the NEBNext Multiplex Oligos for Illumina (Index primer sets A and C) (New England BioLabs) according to the manufacturers instructions. The PCR products were purified using the ZR-96-well DNA Clean-up kit by Zymo Research according to the manufacturers instructions. The DNA quantity was measured with the Qubit dsDNA HS Assay kit (Thermo Fisher Scientific) and samples were pooled in equal amounts of DNA for sequencing on an Illumina MiSeq with 150 bp paired ends.

### Determination of the barcode frequencies

The frequencies of the different barcode in each condition were determined in CLC Genomics workbench (Qiagen) and blasting them against a database composed of the barcode sequences (SI Table 9). A p-value of 0.0001 was used as a cut off for the homology to ensure only perfect matches. A table with the number of hits for each barcode was obtained for each condition and time point. The table was extracted and R was used for further analysis. The counts were normalized to time point 0 and the relative abundance of each barcode was calculated for each condition. Few barcodes from the media adapted lineages and well as DOX adapted lineages could not be detected and were therefore excluded from the analysis. The relative barcode frequencies were plotted over time for each condition using R.

### Fitness calculation based on barcode frequencies

The fitness of the strains was calculated based on the barcode frequencies from the competition experiment and the calculations were performed according to an adjusted procedure by Wetmore *et al*.^6^. The binary logarithm of the number of reads of a specific barcode at time point 0 was subtracted from the number of reads of the same barcode after 8 hours of growth in a specific condition. The average and standard derivation of the triplicates was calculated.

### Statistical tests

The fitness calculated based on the individual growth measurements was used as a predictor variable and the fitness based on the competition experiment served as the response variable for a linear regression model built with ggplot2 in R. The Pearson’s correlation coefficient of determination R^2^ and the p-value were calculated and added to the plots using two functions (stat_poly_eq() and stat_fit_glance()) from the ggpmisc package^50^. Actual p-values can be found in SI Table 10. Following significance levels were used for the p-value in Fig. 5: *for p < 0.05, **for p < 0.001. Moreover, the p-values were validated with an additional spearman’s correlation (using R statsv3.4.4^49^) that is more robust to non-parametric data and can be found in SI Table 10.

### Data availability

The datasets generated during and analyzed during the current study are available from the corresponding author on reasonable request. Barcoded *E*. *coli* strains can be requested from the corresponding author.

## Electronic supplementary material

SI Table 1-10
